# The sonic instructor: A music-based biofeedback system for improving weightlifting technique

**DOI:** 10.1371/journal.pone.0220915

**Published:** 2019-08-28

**Authors:** Valerio Lorenzoni, Jacob Staley, Thierry Marchant, Kelsey E. Onderdijk, Pieter-Jan Maes, Marc Leman

**Affiliations:** 1 Institute for Psychoacoustics and Electronic Music (IPEM), Department of Musicology, Ghent University, Ghent, Belgium; 2 Internet technology and data science lab (IDLAB), Ghent University, Ghent, Belgium; 3 Data analysis Department, Ghent University, Ghent, Belgium; Texas A&M University, UNITED STATES

## Abstract

In this study, we assumed that correct functional movements for weightlifting can be learned with the help of a music-based biofeedback system. We compared musical feedback with verbal feedback from experienced trainers using two independent groups. The focus was on one specific movement called deadlift. Physical parameters under considerations were the spine (i.e. loss of midline stability resulting in flexion) and the forward displacement of the barbell during the repetitions relative to the mid-foot. We recruited 31 recreational weight lifters (21-42 years of age). Results revealed that both feedback types are effective in improving the movements for deadlift. No significant differences were found across the two feedback types, neither in terms of movement, nor in terms of clarity and motivation. The results suggest that the proposed feedback system is a valid tool for technology-aided training and self-training practices.

## Introduction

Biofeedback has been used in the domains of sports [1, 2], motor rehabilitation [3, 4], and even neurological rehabilitation [5]. It provides information about physiological and/or biomechanical parameters related to the performance [6]. Typically, the main goal of such systems is to enable subjects to improve their performances without the explicit instruction of a trainer or therapist. Often biofeedback provides information after the performance of a task. However, due recent technological developments it is possible to provide real-time biofeedback in a continuous way during the performance. Traditionally, the feedback is presented via screens (visual feedback), loudspeaker/headphones (auditory feedback), or vibrotactile devices (tactile feedback) [7, 8]. In this paper, we describe the design and validation of a real-time bio-mechanical auditory feedback system for a weightlifting movement called: *deadlift*.

Researchers have proven the benefits of such functional weightlifting movements [9]. The deadlift is one of the three possibilities in powerlifting. It consists of grasping a barbell from the resting position on the floor, raising the weight by extending the knees, hips, and back, while holding the arms downward. On completion of the movement, the knees must be locked in a straight position and the shoulders retracted. A detailed description of the technique can be found in [10]. There exist a total of eleven variations of deadlift movements as reported by [10, 11]. However, the ones mostly used by athletes are the so called: conventional and nonconventional styles (i.e. sumo). McCuigan *et al*. investigated the biomechanical differences among the last two techniques in [12] and highlighted the advantages of the sumo technique in terms of posture and barbell due to the wider foot stance. In the present study we focus on the conventional style of deadlift, which is mostly used in functional fitness programs (e.g. CrossFit). Due to the fact that the deadlift is a closed chain exercise (i.e. feet in constant contact with the ground), it is often used in the prevention and rehabilitation of anterior cruciate ligament (ACL) reconstruction to improve strength of the muscular structures that surround the knee and hence dynamic stability of the joints. However, improper technique during deadlift lift-off phase (i.e. the beginning of the movement) may predispose the spine and back musculature to an increased risk of injury [13, 14].

Risk of injury is typically related to two main faults in performing the movement, that is, improper spine alignment and barbell path. The fault with respect to the spine is typically a loss of neutrality (i.e. excessive flexion) due to the demands of the external load of the barbell. This creates an undesired outcome by placing a greater proportion of the load on the lower lumbar of the spine, as opposed to being distributed across a range of supporting musculature. The fault with respect to the barbell path has the same potential deleterious consequence, placing a higher demand on the lower lumbar, and increasing the risk of injury. Under proper movement the barbell should travel up the torso while maintaining a vertical line centred over the middle of the foot. Typically, deviation from this path occurs with the barbell drifting over the toes due to the force of the external load. Increased horizontal distance of the barbell relative to the hinge point (i.e. hip) translates to an increase in the moment arm, which once again places higher demands on the lower lumber of the spine, and where the common fault of excessive flexion of the spine occurs.

The presented system aims to provide real-time musical feedback based on sonification of movement performance. The movement quantities being sonified, and on which the participant gets feedback, are the spine curvature and the barbell horizontal displacement, as these quantities are directly related to increased risk of injury. Usually coaches spend time in the first phase to teach the right technique and provide feedback to the performers. However, having continuous feedback by a coach is not feasible while training in public gyms or at home. Therefore there is a need to develop (portable) systems that are able to guide athletes towards the correct movement patterns, thus offering the opportunity to autonomously develop a proper and safe technique.

Sonification of weightlifting movements has been shown to increase average exertion of power compared to silent condition [15]. However, it has not been used for improving the technique, nor for injury prevention. In our study, sonification is meant as a manner of steering the correctness of the movement without explicit indication by verbal cues from a human trainer, thereby laying the ground for safe movement habits and thus for injury prevention. Our steering paradigm is based on reinforcement learning, the idea that subjects learn in order to maximise the reward of the outcome of their actions, and minimise the punishment due to unwanted outcomes of their actions. When coupling reward and punishment to a desired behavior, subjects are likely to learn to exhibit this behavior spontaneously, without needing to be told explicitly what to do. Reinforcement learning thus steers subjects’ behaviour without telling how to act. Thereby, subjects have to find the proper solution for achieving the correct performance.

In this context, music is particularly relevant as it may be a rich source of auditory reward and pleasure [16, 17]. Music was shown by several researchers to have positive effects during physical activities: enhance work output [18, 19] increase arousal [20], and boost mood states [21, 22], especially when music is synchronised with the tempo of movement. In the present study, we use music and its sound quality as reward, whereas we use distorted music, that is, music with bad sound quality as punishment. Obviously, reward (through increasing the sound quality) is associated with correct movements, whereas punishment (through decreasing the sound quality) is associated with incorrect movements. More specifically, the unwanted movements (spine forward bending and barbell forward displacement with respect to initial position) cause a down-sampling of the played music track and a forward panning (and reduction in number) of the active loudspeakers.

Our hypothesis is that a music-based feedback system, working with reinforcement learning, could be comparable to the verbal instructions of a human instructor. We also assume that the sonified feedback would be more motivating than standard verbal instructions due to the reward mechanism and pleasurable effects of music. Participants were split into two groups upon arrival. The groups were balanced as much as possible in terms of sex and experience. One group received only verbal feedback and the other group only sonic feedback during 10 deadlift repetitions. We compared the movement parameter after feedback with a control condition without any feedback, taken as reference of the participant’s initial movement patterns. To test our hypothesis the movement improvements with respect to the control between the two groups were compared.

## Materials and methods

### Participants

Recruitment of the participants started about two months prior to the tests by advertisement in local gyms and crossfit centres. Publicity was also made at the entrance of De Krook library in Ghent, where the laboratory is located. Thirty-one participants (14 female) took part in the experiment. The age range was 20 to 42 years (mean = 29.2, sd = 5.7).

All the participants were trained in sports. In particular, 11 participants mentioned to have more than 2 years of experience with deadlift movements, 15 between 6 months and 2 years and 5 declared to have less than 6 months of experience with it. The majority of participants (19) declared to mostly use music while training, 6 to train without music and 6 equally with and without music. Fourteen participants (45%) declared to have received music education in their life.

The study was approved by the Ethics Committee of the Faculty of Arts and Philosophy of Ghent University, and all procedures followed were in accordance with the statements of the Declaration of Helsinki. All participants voluntarily participated. They were informed about the physical effort required for the experiment and that questionnaires could have contained personal questions. As compensation, participants received a voucher for a consumption at the library restaurant.

### Apparatus

**Fig 1:**
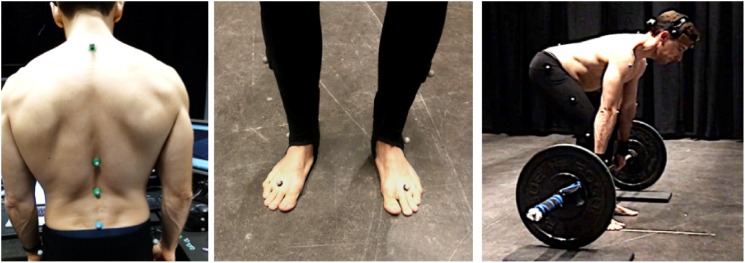
Mocap markers positioning.

The experiment took place at the IPEM-IDLab Art&Science Lab in De Krook library in Ghent. The laboratory has dimensions: 10 m x 10 m x 7 m height and is instrumented with an immersive sound system of 64 speakers Martin Audio CDD6, distributed all around the room (two circles at different heights and on the ceiling). A male barbell (20 kg) with 2 x 5 kg weights was placed at the center of the room on two protective hard foam pads. Eight Qualisys infrared cameras were used for the motion capture (Mocap) recordings. The cameras detected passive reflective spherical markers of 2.5 mm radius. Participants were equipped with a full-body markers set-up, consisting of a total number of 22 markers. Trousers with a fixed configuration of 6 markers were provided to the participants (two different sizes were made available). Two straps with a single marker were placed on the wrists; a headset with 4 fixed markers and adjustable width was used for the head. A total of 10 markers were attached directly on the skin of the participant, using a biocompatible double sided tape by 3M. Specifically, 4 markers were attached on the spine at the height of the vertebras: L4, T12, T7 and C2. The larger spacing between the last two markers was chosen to account for the presence of sport tops used by female participants. Markers were also attached on the elbows, shoulders, and on the front part of the feet. Four markers were placed on the barbell: two at the extremities and two on the clap next to the weight on one side only. Asymmetry was chosen to improve barbell model recognition. See images in Fig 1 for visualization of the full positioning of the markers. The subject in the pictures is one of the coauthors and has given written informed consent (as outlined in PLOS consent form) to publish these pictures.

Mocap recordings were performed on a dedicated Windows computer. The system evaluated the 3D markers positions at a frequency of 100 Hz. These position were transmitted in real-time as OSC message to a custom-build Max4Live patch implemented as audio effect within Ableton Live. The Max4Live patch was responsible for starting and stopping the music, providing the sonification based on the physical parameters, and storing the data.

The Max4Live patch calculated the following quantities, used for both analysis and sonification.

**Spine bend**. The sum of the consecutive euclidean distances between the four spine markers: *d*0 + *d*1 + *d*2. This length is directly linked to the spine bending, which is the physical parameter we tried to optimize for injury minimization, and therefore named spine bend.
**Barbell-foot (B-F) distance**. The horizontal component of the distance between the line connecting the extremities of the barbell and the markers on the front part of the feet. The smaller of the two distances was considered.

**Fig 2:**
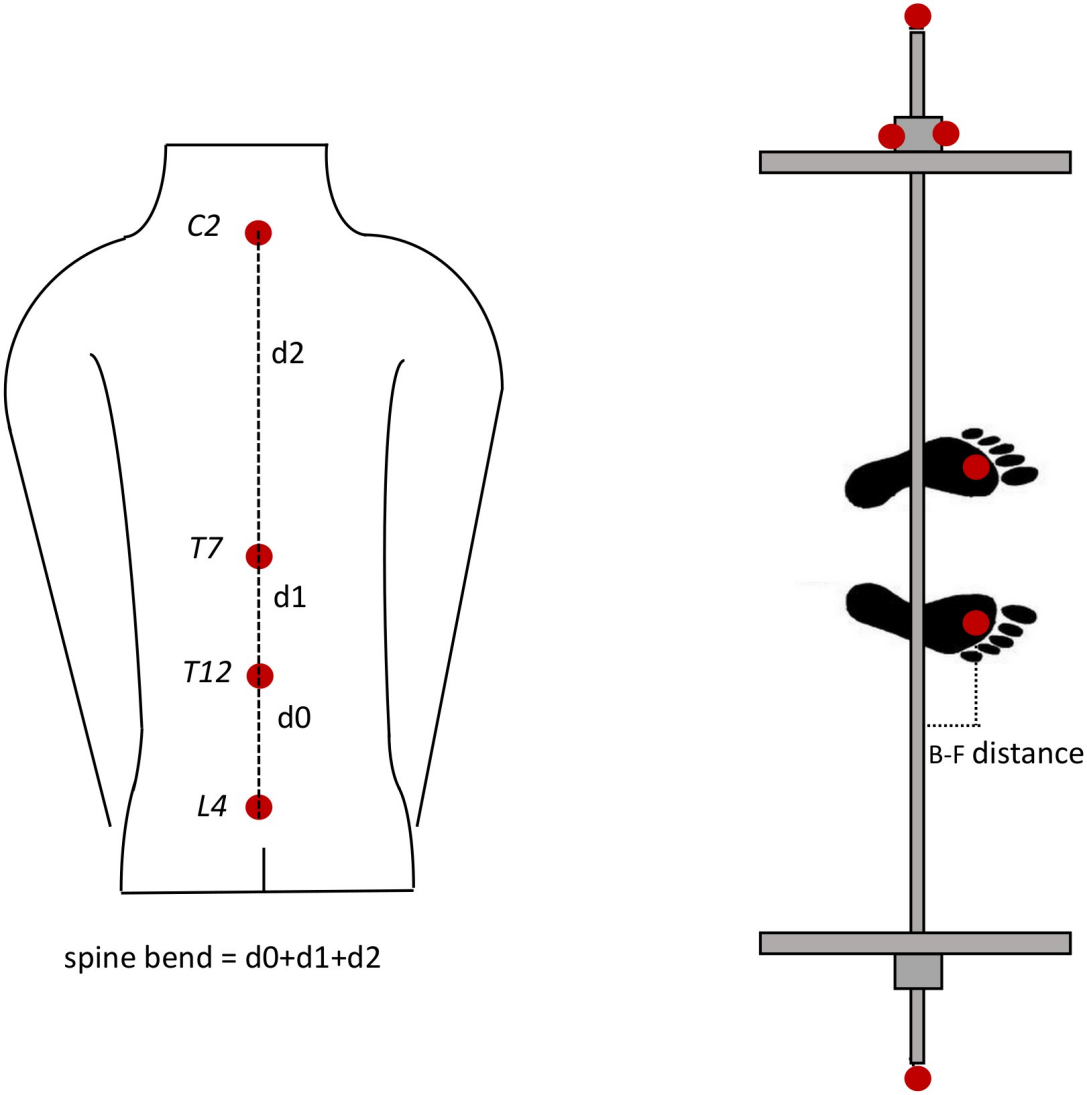
Sketch of the derived measured quantities: Spine bend (left) and barbell-foot distance (right).

See Fig 2 for a schematic visualization of the above quantities.

### Experimental procedure

Once in the lab, participants received a written description of the experiment. They were asked to sign an informed consent form and fill in a questionnaire with general information about sex, age, level of experience with weightlifting, music education, injuries. Subsequently, a video was shown of an expert performing 10 deadlifts, in front and side view. It was explained that the focus of the experiment was on the neutrality of the spine during the movements and verticality of the barbell path.

Participants were then equipped with the markers set-up. The Qualisys software made use of a pre-trained skeleton model to recognize the body parts across different participants. A separated model for the barbell was used. The correct labelling of the markers was checked at this stage.

One of the authors is a certified Level 2 Crossfit trainer and functioned as the instructor during the experiments. A warm-up routine was provided by the instructor to all participants prior to the tests. At this point reference parameters for each subject were recorded.

**Neutral spine**. Participants were asked to grab the bar standing and keep the back in unloaded neutral position.
**Max spine bend**. Participants were asked to grab the bar on the ground and slightly bend the back upwards. The instructor helped them find this incorrect position, corresponding to the maximal deviation from target movement.
**Initial B-F distance**. Participants were asked to grab the barbell on the ground as if they would start the movement and place it approximately in the middle of the foot. The initial (horizontal component of the) distance between the barbell and the feet was then recorded.

Based on these values the Max4Live patch calculated in real-time the following non-dimensional quantities, which were used for the analysis:

- non-dimensional spine bend (*sb*),
  $$\begin{array}{c} sb=\frac{spine\;bend-neutral\;spine}{max\;spine\;bend-neutral\;spine} \end{array},$$
- non-dimensional barbell-foot distance (*bfd*),
  $$\begin{array}{c} bfd=\frac{BF\;distance}{initial\;BF\;distance} \end{array}.$$

Participants were informed that, to perform a correct movement, the spine would have to remain in the measured neutral position throughout the movement and that the barbell would have to remain at the same distance from the toes as measured by Initial B-F distance to ensure verticality of the barbell path.

The actual tests started with a serie of 10 deadlifts at own tempo, middle-low pace. This was taken as *control condition* for the analysis. Subsequently, participants were split into two groups, as homogeneous as possible in terms of sex and experience level. Experience level was based on the information provided by participants at the beginning of the session while compiling a general questions form. Participants were requested to select among three options about experience level: “less than 6 months”, “between 6 months and 2 years”, “more than 2 year”. These were labeled as level A, B and C, respectively and used as ordered factors in the analysis. Specifically, the instruction group consisted of 2 level A participants (1 male and 1 female), 9 level B (4 female and 5 male), and 5 level C participants (2 female and 3 male). The sonification group consisted of 3 level A participants (3 male), 6 group level B (4 female and 2 male), and 6 level C participants (3 female and 3 male). Posterior analysis of homogeneity of these parameters among the groups by *χ*^2^ confirmed homogeneity (*χ*^2^(2) = 1.778, *p* = .411). One group of participants received verbal feedback by the instructor, while the other group received sonification as feedback. The groups are hereafter called *instruction group* and *sonification group*, respectively.

Participants of both groups were asked to perform 10 deadlifts for each of the following points of performance: *spine*, *barbell* and *combination*. The points of performance were chosen to ensure the participant performed the movement with safety and efficacy in mind. The first two points of performance were randomized across participants, while the *combination* was always last. Participants were instructed to only focus on the specific performance point, and informed that continuous feedback (either as verbal instruction or as sonification) would be given only if the movement deviated from correctness for the specific point of performance under analysis.

In particular, concerning the spine, feedback was only provided if the spine curvature was positive (i.e. increased distance between spine markers, *sb* larger than zero), meaning forward bending, as this is directly responsible for increasing load on lower lumbar spine. Concerning the barbell-foot distance, feedback was only provided if the barbell moved towards the toes during the movement (i.e. *bfd* approaching zero) as this would increase the momentum on the back and increase the risk of injuries.

Hereafter, the explanations provided to participants, respectively for the *instruction group* and the *sonification group* and for the specific point of performance, are reported.

#### Instruction feedback

In this case the instructor informed the participants as follows.

*Spine*: “You will receive feedback when your spine deviates from the neutral starting position. You will focus on maintaining your neutral spine position during the movement, as well as keep your shoulders retracted. The two cues you will hear are ‘straight spine’ and ‘shoulders back’ as an instruction to correct these faults.”
*Barbell*: “You will receive feedback when the bar path deviates from the vertical line of your mid-foot. You will focus on maintaining a vertical path of the bar throughout the movement by maintaining a vertical shin and keeping the bar close to your shins, thighs and torso during the progression of the movement. If the horizontal distance of the bar from your body increases, the cue you will hear to correct this is to ‘keep the bar close’, which is a reminder to engage the lats and force the barbell back to the body. The second cue will be ‘knees back’, which is instruction to drive your knees back to create vertical shins.”
*Combination*: “You will receive feedback on both points-of-performance, which are correct spine position and barbell path. The cues you will hear will be the same as before.”

#### Sonification feedback

The feedback in this case consisted of modifications of the base music track if the movement deviated from correctness.

*Spine*: “The music will be distorted when your spine curvature deviates from the initial neutral position we measured. Try to improve the music quality by improving your spine curvature. You can hear the effect if you try to bend your spine.”
*Barbell*: “The audio configuration will change if the barbell path deviates from verticality. Try to improve the music quality by focusing on the barbell path. You can hear the effect if you try to move the barbell towards the toes.”
*Combination*: “This is a combination of the previous two audio modifications. Try to pay attention to both parameters.”

After each series of repetitions, a break of 5 minutes was introduced to enable the participant to recover sufficiently. During the break they were asked to fill out a Rating of Perceived Exertion (RPE) questionnaire [23] and indicate how heavy the effort had been during the exercise, ranging from 6 (“no exertion at all”) to 20 (“maximal exertion”). In order to test the differences in motivational properties of the feedback, participants also performed a modified version of the Brunel Music Rating Inventory 2 (BMRI-2) test [24]. In this test, they were asked to rate on a 7-point Likert scale: clarity, pleasantness, accuracy, motivational properties, and usability of the presented audio feedback. All questionnaires were implemented as Google forms on a dedicated Apple computer within the same room, only used by the participants.

### Stimuli

#### Music

In all conditions, the same music track was played. The piece was specifically composed for this experiment by the authors. The music was composed respecting the following requirements:

- to be unknown, to avoid personal affection,
- to be instrumental (no lyrics), to avoid focus on content,
- to have a clear beat, to stimulate repetitive movements.

#### Sonification

The sonification group received feedback on their movement performance through alterations of the baseline music track. Alterations were based on a non-linear mapping of the input physical parameters, according to the following logistic function:

$$\begin{array}{c} y=1-\frac{1}{1+e^{-\alpha *(x-\beta ))}} \end{array}$$ (1)

where *x* represents the physical variable to be mapped and *α* and *β* empirical parameters determined in a preliminary testing phase to ensure enough responsiveness of the feedback and margin for movement execution without distortion. More specifically, the implemented feedback mechanisms consisted of:

**Spine bend feedback**. Continuous variations of the sampling rate of the music track.
  In practice, the non-dimensional spine bend value *sb* was mapped into the logistic function of Eq 1 with parameters: *α* = 10 and *β* = 0.2. The resulting output value (comprised between 1 and 0) provided the input of the *degrade*_~_ object implemented in Max4Live. This corresponded to alterations of the actual sampling size of the audio buffer, resulting in a “metallic sounding” distortion.
**Barbell-foot distance feedback**. Change in panning and number of effective speakers.
  The *bfd* was mapped into Eq 1 with parameters: *α* = 15 and *β* = 0.6. The output *y* in this case determined the gain of the Ableton master track right and left output channels, in counterbalanced order. The two stereo channels outputs of the Ableton Master track were mapped to different speaker configuration using the Dante Controller software by Audinate. The right channel was mapped to a speaker configuration surrounding the participant, while the left channel was mapped to only the 3 speakers in front of the participant. Consequently, if the value *bfd* moved towards 0 (i.e. barbell toward the foot) the gain of the left channel (linked to the three speakers in front only) was increased while that of the right channel (linked to the all surrounding speakers) was decreased. This implied volume reduction and directionality change, giving the participant the feeling that sound was only coming from the front.

The music track together with the Max4Live patches for music alterations, can be found and downloaded as Ableton project at the following repository: https://doi.org/10.5281/zenodo.3355107.

### Data acquisition

The markers’ positions were acquired by the Mocap system on the Mocap computer and streamed in real-time as OSC to the Max4Live patch on the control computer. The derived quantities *sb* and *bfd* were calculated by the same patch. Recordings were made every 10 milliseconds and stored as .txt file on the Windows computer running Ableton. The stored files contained the following quantities:

- 3D markers’ positions
- *sb* and *bfd*
- distortion level (only for sonification feedback)
- right and left output channel volume (only for sonification feedback)

A separate file was stored for each participant and each point of performance. The questionnaire information was directly put into Google Drive.

### Data analysis

The series of repetitions were split into single deadlift movements by analysis of the vertical barbell displacement using a custom Python script. The first three and last two repetitions were discarded in the analysis to minimize transient effects. One mean value of the measured quantities over five deadlift repetitions was calculated for each participant and each condition.

Our hypothesis is that our music-based auditory feedback would perform comparably to human instruction. To prove our hypothesis, the mean changes in movement with respect to the control condition after the feedback by the instructor and after the sonic feedback, were compared. More specifically, differences between the means of spine bend (*sb*) and barbell-foot distances (*bfd*) in the feedback condition and control condition (feedback—control) were evaluated for the instruction and sonification groups separately.

We hereafter refer to as “improvement in spine bending”, a reduction of the mean *sb* with respect to the mean *sb* in the control condition, which corresponds in practice to a decrease in spine forward bending. An “improvement in barbell-foot distance” is defined as an increased average distance of the barbell from the mid-foot during the feedback session compared to the control session. This indicates that the barbell travels a mean vertical path during the movement which is closer to the body compared to the control condition.

Statistical analysis using the software RStudio 1.0.136, was performed on the differences between the instruction and sonification groups and for the different points of performance. Questionnaires information about clarity and motivational properties of the feedback for each point of performance were also analyzed.

## Results

### Performance distributions

#### Spine bend differences

**Fig 3:**
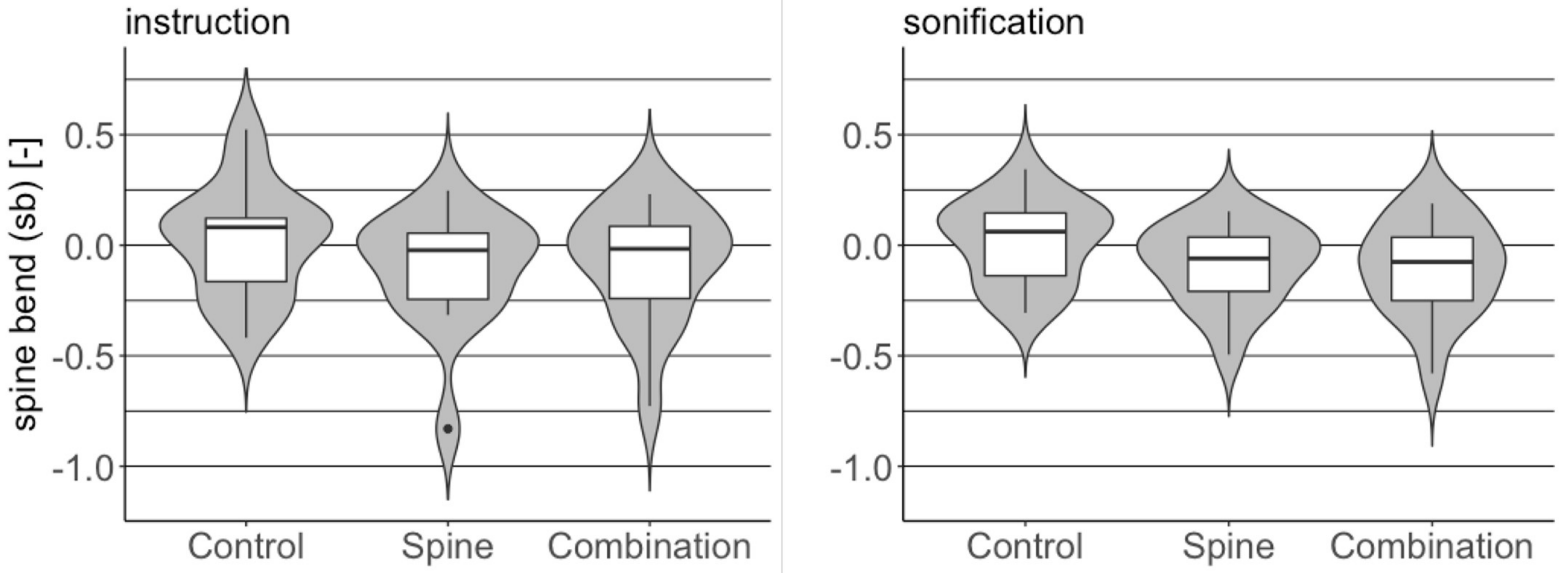
Non-dimensional spine bend distributions for points of performance *spine* and *combination* and control condition. Instruction group (left plot) and sonification group (right plot).

Fig 3 shows the distributions of mean performances across participants for instruction (left) and sonification (right) feedback. In this case, the shown points of performances are: *spine* and *combination*, for which participants received feedback on the spine curvature. A value 0 indicates that mean spine curvature corresponded to neutral position throughout the deadlift repetitions. Negative values indicate backward extension and positive values indicate forward bending. The latter was corrected through feedback.

The values of the distributions of non-dimensional spine bend for all points of performance and control are reported in Table 1. Means and standard deviations are reported for the normally distributed variables, medians for the non-normally distributed ones. Results of statistical tests against the control condition are also reported. For the normally distributed variables t-test are used, t-values and significance *p* are reported. For non-normally distributed variables Wilcoxon tests were used, z-values and significance *p* are reported.

**Table 1:** Distributions of non-dimensional spine bend for all points of performance and control. For the normally distributed variables, means, standard deviations, t-values, and significance p are reported. Medians, z-values and significance p values for the non-normally distributed ones.

	**instruction**		**control**		**test statistics**	**p**
	mean	sd	mean	sd		
spine	-0.022 (Mdn)	–	0.021	0.253	z = -0.580	0.0001
barbell	0.069	0.441			t = 0.541	0.597
combination	-0.087	0.262			t = -4.306	0.0007
	**sonification**		**control**		**test statistics**	**p**
	mean	sd	mean	sd		
spine	-0.888	0.18	0.017	0.187	t = -2.651	0.018
barbell	-0.013	0.191			t = -1.451	0.167
combination	-0.106	0.211			t = -3.275	0.005

Comparisons of point of performance *spine* and *combination* with the control condition yield significant differences for both instruction (*p* = .0001 and *p* = .0007 for *spine* and *combination*, respectively) and sonification feedback (*p* = .018 and *p* = .005 for *spine* and *combination*, respectively). As expected, no significant difference with respect to control is observed for point of performance barbell as no feedback on spine bend is provided in the session for this point of performance.

#### Barbell-foot distance differences

**Fig 4:**
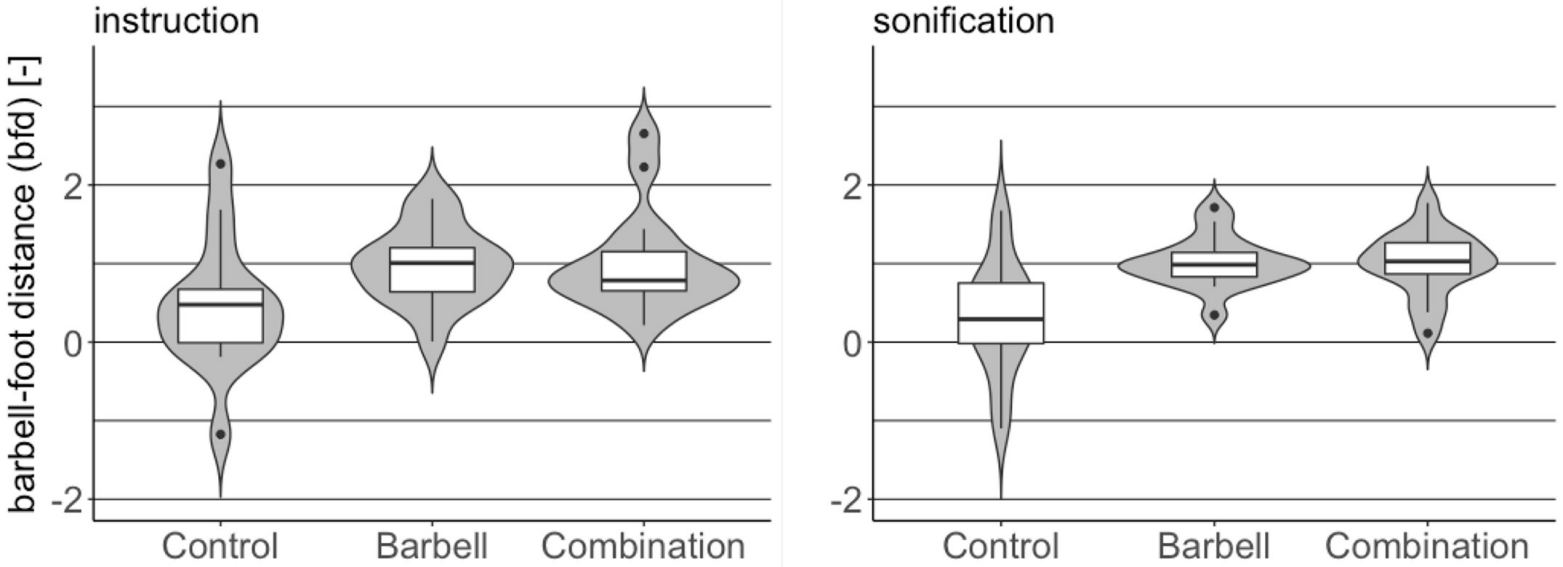
Non-dimensional barbell-foot distance distributions for points of performance *spine* and *combination* and control condition. Instruction group (left plot) and sonification group (right plot).

The distributions of the *bfd* are shown in Fig 4 and summarized in Table 2. In this case a value 1 indicates that the mean barbell-foot distance was equal to the initial measured barbell-foot distance throughout the deadlift repetitions. Values larger than 1 indicate larger mean barbell-foot distance than the initially measured distance and negative values indicate mean barbell horizontal position beyond the front-foot markers. The latter was corrected through feedback.

**Table 2:** Distributions of non-dimensional barbell-foot distance for all points of performance and control. For the normally distributed variables, means, standard deviations, t-values, and significance p are reported. Medians, z-values, and significance p values for the non-normally distributed ones.

	**instruction**		**control**		**test statistics**	**p**
	mean	sd	mean	sd		
spine	0.54	0.827	0.455	0.824	t = 0.972	0.346
barbell	0.973	0.482			t = 3.546	0.003
combination	1.028	0.656			t = 3.044	0.008
	**sonification**		**control**		**test statistics**	**p**
	mean	sd	mean	sd		
spine	0.47	0.687	0.329	0.72	t = 1.920	0.073
barbell	1.014	0.324			t = 3.802	0.002
combination	1.027 (Mdn)				z = -3.522	0.0004

Comparisons of barbell-foot distance for points of performance *barbell* and *combination* with the control condition yield significant differences for both instruction and sonification feedback. In this case, no significant difference with respect to control is observed for point of performance *spine* as no feedback on barbell-foot distance was provided in the session for this point of performance.

### Difference between feedback types

In order to compare performances between the feedback types, movement improvements were considered. These were calculated as the differences of the mean measured output before (control condition) and after the feedback, for the two groups (instruction and sonification) separately. Only the point of performance *combination* is considered, as that is representative of the total final movement improvement. The same analysis was performed for the spine bend (sb) and barbell-foot distance (bfd), separately.

#### Spine bend differences

A preliminary analysis of normality of the data using Shapiro-Wilk test showed that the means of non-dimensional spine bend *sb* feedback—control was normally distributed for both feedback types: instruction *p* = .091 and sonification *p* = .068 and for the controls of both groups, control (sonification) *p* = .752 and control (instruction), *p* = .690. Therefore parametric tests were used for the comparisons. The results of the comparisons are shown in Table 3.

**Table 3:** Comparisons of non-dimensional spine bend for point of performance *combination* of both feedback (*instruction* and *sonification*) with respect to the control condition and between each other. Significance is highlighted with an asterisk (*).

spine bend (sb)		test statistics	p
control (instruction)	control (sonification)	t = -0.037	0.971
instruction	control (instruction)	t = -4.306	0.0007*
sonification	control (sonification)	t = -3.275	0.005*
instruction	sonification	t = -0.226	0.822

From the first row of Table 3 it can be observed that there is no significant difference between the two groups in the control phase. This confirms that initial movement parameters between the groups are statistically equivalent. Comparison of the spine bending between the control condition and the feedback reveals significant differences (*p* = .0007 and *p* = .005 for the instruction and sonification feedback respectively), indicating that both feedback types are effective in modifying spine bending. No significant differences could be observed in average spine bend performances between the two feedback types (row 4 of Table 3, *p* = .82).

**Fig 5:**
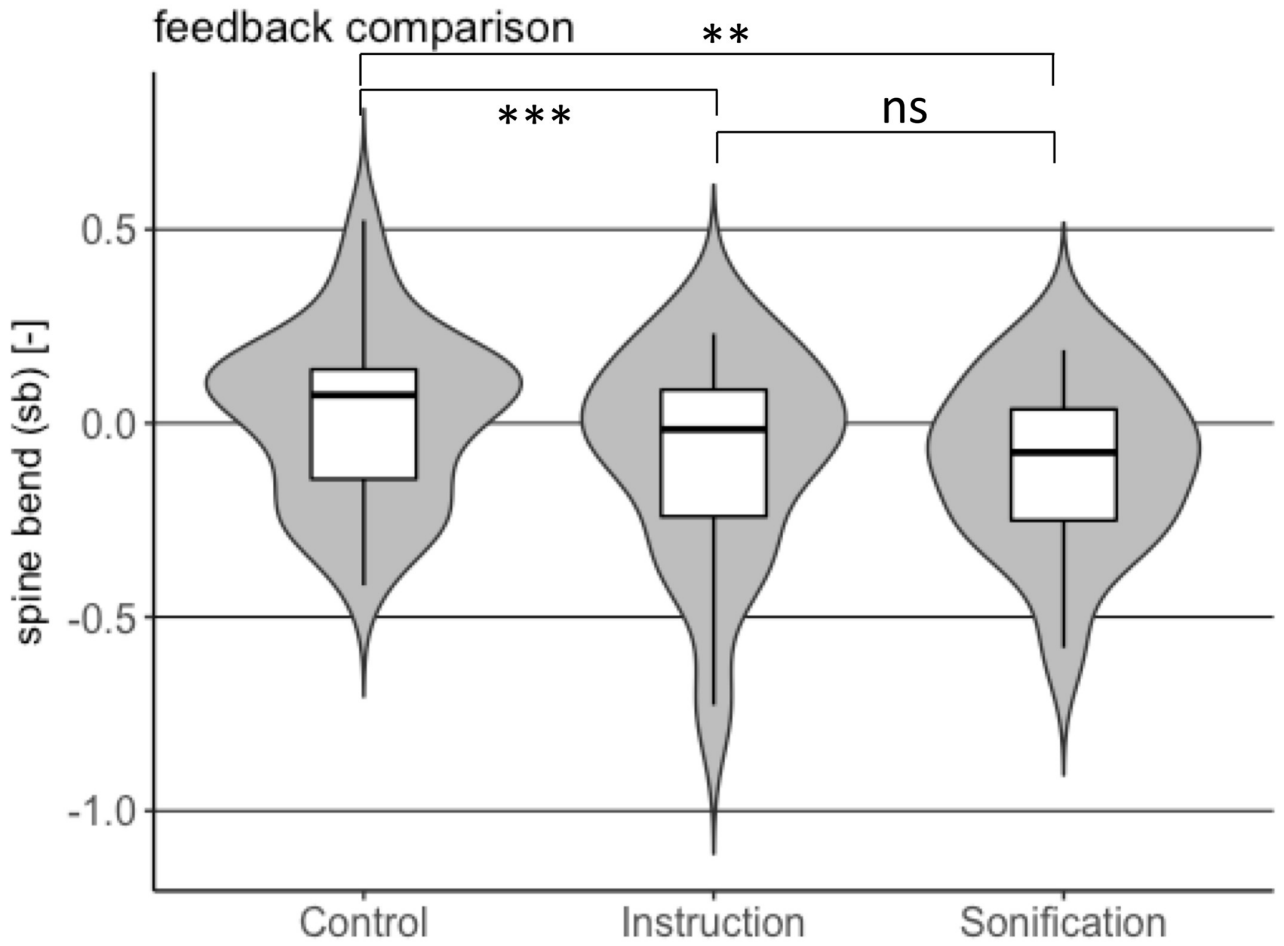
Comparison of spine bend distribution between instruction and sonification feedback with respect to control for point of performance *combination*.

Distribution of spine bend for point of performance *combination* for the two feedback types and control are shown in the violin plots of Fig 5.

#### Barbell-foot distance differences

Tests of normality for the barbell-foot distance improvements (*bfd* feedback—control) by Shapiro-Wilk revealed that the differences between control of both groups are normally distributed, *p* = .398 for instruction and *p* = .153 for sonification group respectively. The movement improvement for the instruction group is also normally distributed (*p* = 0.316), while for the sonification group the barbell foot improvement is not normally distributed (*p* = 0.037). Parametric t-tests were used for the normally distributed differences while Wilcoxon Rank Sum tests for the non-normally distributed pairs. Results of the comparisons are shown in Table 4.

**Table 4:** Comparisons of non-dimensional barbell-foot distance for point of performance *combination* of both feedback types (*instruction* and *sonification*) with respect to the control condition and between each other. Significance is highlighted with an asterisk (*).

barbell-foot distance (bfd)		test statistics	p
control (instruction)	control (sonification)	t = -0.451	0.654
instruction	control (instruction)	t = 3.044	0.009*
sonification	control (sonification)	z = -3.522	0.0004*
instruction	sonification	z = -0.683	0.805

**Fig 6:**
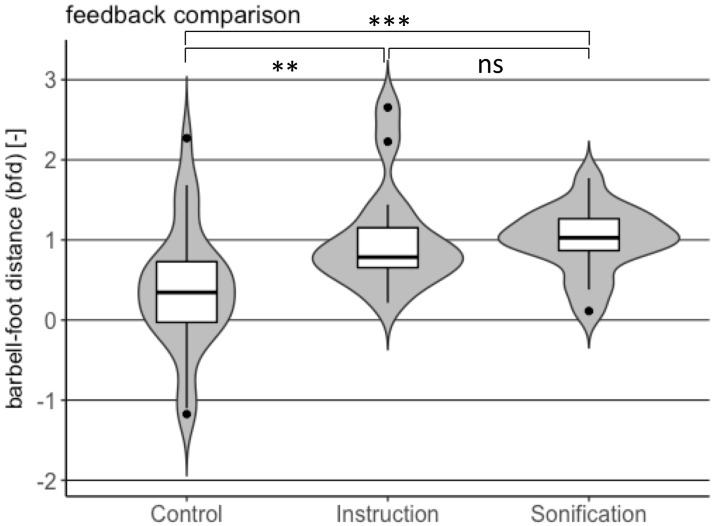
Comparison of barbell-foot distance distribution between instruction and sonification feedback with respect to control for point of performance *combination*.

Distributions of non-dimensional spine bend and non-dimensional barbell-foot distance in point of performance *combination* for the two feedback types and control are shown in the violin plots of Figs 5 and 6, respectively.

The main result of the presented analysis is that for both feedback types there is a significant movement improvement.

#### Improvements comparison

From Table 5 it can be observed that the movement improvements with respect to control do not significantly differ for the two feedback types, neither for spine bend (*p* = .721) nor for barbell-foot distance (*p* = .922) This result seems to demonstrate that both feedback types are statistically non-significantly different from each other to achieve movement improvement. In particular, for the spine bend variable, the instruction feedback produces a mean improvement of -0.108, while this is -0.124 for sonification. For the barbell-foot distance the instruction feedback produces a mean increase of 0.573, while sonification a median increase of 0.755.

**Table 5:** Comparisons of movement improvements between feedback types (instruction and sonification) for point of performance *combination*. Non-dimensional spine bend improvement and non-dimensional barbell-foot distance on first and second row respectively. Means, standard deviation and t-values are reported for normally distributed quantities, medians and z-values for non-normally distributed ones.

	movement improvement		comparison	
	instruction—control	sonification—control	test statistics	*p*
	mean (sd)	mean (sd)		
**spine bend**	-0.108 (0.096)	-0.124 (0.152)	t = -0.362	0.721
**barbell-foot distance**	0.573 (0.729)	0.755 (Mdn)	z = -0.097	0.922

### Effect of expertise

Tests were performed to check if the level of expertise of the participants had an influence on the movement improvements by the feedback. Linear models were constructed using movement improvement as independent variable and two fixed effects, namely: feedback type and level of expertise. Four versions of this model were considered, varying the set of predictors: the null model (model 0, without feedback type and expertise), the full model (model FULL including predictors: feedback type and expertise), and two intermediate models (model FB with only feedback and model EXP with only expertise). The models were compared by means of ANOVA using the standard “anova” function implemented in R. The same models were constructed considering movement improvements of spine bend and barbell-foot distance, respectively. Results of the comparisons for spine bending models revealed that nor feedback type nor level of expertise have an effect on movement improvement. Comparison of model 0 with models FULL, FB and EXP yielded non-significant differences, respectively *p* = .635, .723 and .431. Comparisons for the barbell-foot distance variable produced the following results: model 0 with model FBD, *p* = .701, model 0 with model EXP *p* = .025* and model 0 with model FULL *p* = 0.065. From the last results, it can be seen that experience is indeed a predictor of barbell-foot distance improvement while feedback is not. The predictions of model EXP for barbell-foot distance improvements are: 1.29 for group A, 0.376 for group B, and 0.67 for group C. These results indicate that beginners of group A are the ones featuring largest improvement for barbell verticality. Surprisingly, the intermediate participants of group B featured less improvement compared to the experts of group C.

### Questionnaires information

After each set of repetitions, participants were requested to fill in a questionnaire describing the perceived feedback. The asked questions concerned the following characteristics, in order: Perceived Effort, Clarity of feedback, Pleasantness, Motivation, Accuracy, Match between performed movement and perceived feedback, and Usability of the system. Answers were provided on a 7-point Likert scale from 1 to 7. Participants could further indicate if they received no feedback at all by inserting 0. The questionnaires information relative to two participants of the instruction group could not be used due to incomplete answers and were disregarded in the analysis. For the instruction group, 13 trials relative to a total of 8 participants received no feedback. The trials included all different points of performance (5 for *spine*, 4 for *barbell*, and 4 for *combination*). This indicates that movement was correct according to the trainer and no feedback was provided. For the sonification group, only 4 trials received no feedback, all relative to point of performance *spine*. Screenshots of all questionnaires can also be found at: https://doi.org/10.5281/zenodo.3355107.

#### Differences between feedback types

Comparison of ratings of the two different feedback types was performed using Mann Whitney U tests. No significant differences were found between the two feedback types for the following characteristics: Perceived Effort *p* = .216, Clarity of feedback *p* = .634, Pleasantness *p* = .729, Motivation *p* = .853, Accuracy *p* = .838, Match between performed movement and perceived feedback *p* = .253 and Usability *p* = .224). This indicates that the proposed sonification feedback is performing analogously to standard instruction in terms of the above mentioned characteristics.

Correlation of the improvement with feedback ratings was performed using Kendall’s tau-b correlation tests. No correlation was found between the movement improvement and the asked characteristics of the feedback. This indicates that, in the present case, a better perception of the feedback did not directly translate into movement improvement. Within the same feedback group, no significant differences in terms of feedback characteristics were found across the different points of performance. Music background of the participant within the sonification group did not play a significant role in terms of movement improvement. Comparison by Mann Whitney U tests of the participants with music education (6) compared to the ones without (10) within the sonification group, yielded *p* = .382 for the spine bending and *p* = .635 for the barbell-foot distance. The latter results suggest that music education did not play a role in terms of movement improvement neither for the spine bending nor for the barbell-foot distance.

## Discussion

In this study, we assumed that correct functional movements for weight lifting can be learned with the help of a music-based biofeedback system. We focused on one specific movement called deadlift, and we compared musical feedback with verbal feedback by an experienced trainer. Our hypothesis was (i) that musical feedback has an effect on measured movements of the deadlift similar to verbal feedback, and (ii) that the musical feedback would be more motivating than the verbal feedback. The rationale was that music works as a natural reward, and therefore, that its sound quality can be used as a stimulus for reinforcement learning.

The test of the first hypothesis was based on an independent group analysis of measured parameters of the deadlift movement, namely spine bend and barbell-foot distance, before and after receiving feedback. No significant differences in performances were found. This confirms the first hypothesis, suggesting that the response of a weight lifter, either to a music-based biofeedback system or to a trainer’s verbal instructions, are similar. This result opens for the possibility of using music-based biofeedback systems in the field of weightlifting. Apart from learning the technique, the system could be used by advanced athletes to further improve their technique by discovering minor aspects of their movement that are not fully visible by eye. In using the music-based biofeedback system, several participants reported discovering details about their movements they were not aware of.

The test of the second hypothesis was based on questionnaires. Although the musical feedback scored high regarding clarity and pleasantness, no significant difference in results could be observed when compared against the instructions from the human trainer. This can be ascribed to the fact that most athletes are more accustomed to human feedback while performing sports. In addition, it should be mentioned that the feedback strategy was based on alterations of one basic music soundtrack chosen by the researchers rather than by the weightlifters themselves. Familiarity with the music, as well as the possibility of choosing one’s own music, may have been a determining factor. In this study, music was used as a regulator of reward. We thereby focused more on modifying the sound quality of a given music, rather than choosing the optimal preferred or familiar music. We reasoned that good sound quality of the music could work as reward and that bad sound quality of the same music could work as punishment. Through sonification, i.e. the translation of movement into sound quality, it then becomes possible to influence the functional movements of the weight lifter. Good movements are thereby translated into good sound quality, while bad movements are translated into bad sound quality. Given the fact that music is a natural reward, then it follows that good versus bad sound quality can steer actions towards obtaining the reward. More specifically, reinforcement can steer functional movements of the deadlift towards the intended correct and safe movements.

Movements driven by musical feedback tend to be more correct than movements that are not driven by musical feedback (the control condition). The effect of musical feedback is similar to the effect of verbal feedback. Results thus showed that the proposed feedback system is able to improve the movement technique compared to the control condition and that there is no difference between the verbal and sonification feedback. Applications of reinforcement learning has proven to be effective in other sports applications (see [25]) as long as the subjects are able to modify their behavior and achieve reward. In contrary, a negative reinforcement was observed when participants were not able to achieve the target behavior. This could be partly explained by the fact that the use of such a technological system is relatively new and would need time for athletes to get acquainted with. It seems that participants receiving the musical feedback were more focused on minimising music alterations, than on the motivating value of the music itself. In future experiments, different signification strategies could be compared as feedback, and the effect of different background music tracks could also be investigated.

A positive correlation was found between level of improvement and experience level of the participant for the barbell-foot distance, irrespective of the feedback kind. In particular, the least experienced participants featured the highest improvement. Surprisingly, the intermediate participant group featured lower movement improvements with respect to the expert group. This could be explained by the fact that experts are experimenting more with the system compared to intermediate experienced participants. However, larger group with similar expertise levels would be required to draw strong conclusions on this aspect.

In the present experiment, feedback of the spine curvature was only provided for positive spine curvature, i.e. flexion (forward bending), and no feedback was provided for extension (backward bending). The reason for this was two-fold. The first was that this is typically the main fault in deadlift, and where the majority of injuries arise; excessive flexion of the spine tend to occur especially when weights increase. This can arise from an improper starting position, which impacts the rest of the movement, or a loss of neutral spine (i.e. flexion) during the progression of the movement due to the force of the external load. The second reason was that providing feedback also on extension was deemed to be excessive and to overload the participants with too much information that would not be actionable in correcting the main concern, i.e. forward bending of the spine under load.

The same barbell (20kg) and weights (two 5 kg plates) for a combined weight of 30kg were used for the experiment for all participants. No effect of fatigue was reported by any of the participants, which all reported to regularly perform sport activities.

Previous works by Murgia *et al*. [15] and Fritz *et al*. [26] have focused on the increase in power output and arousal deriving from sonification of movements in weightlifting. The focus of the present work was more oriented towards improvement of technique for injury prevention, and power output was not directly measured. The authors believe that the empowerment deriving from music-to-movement alignment might lead to alterations of movement patterns and particular attention should be paid to pure sonification of movements in sports that require specific technique for minimizing the risk of injuries. Future tests might be dedicated to investigating the effect of the presented system on power output.

Each biofeedback method appeared to result in moderate to large effects immediately during application. However, it is unknown whether the effects were maintained after the experimental session (i.e. in future workout sessions). Future studies should include retention tests to assess the long-term success of the proposed biofeedback method.

The present approach is tailored to the single participant in terms of correct and dangerous positions. An alternative procedure for automatic classification of movement correctness is currently being developed; this is based on machine learning algorithms based on movement acquisitions on a large panel of individuals. Such procedure would generalize the movement parameters and minimize inter-subject variability when acquiring the initial parameters.

Further improvements of the system could derive from increasing portability, e.g. by using wearable sensors for back posture detection. The proposed audio modifications could be easily implemented on portable music players and headphones. This would allow the system to be used in more ecological settings and by multiple athletes simultaneously. Furthermore, the proposed feedback approach could be extended to other functional movements to address similar challenges for improving technique and safety.

### Conclusions

A music-based biofeedback system was developed that is able to provide auditory feedback when performing deadlift movements. The system is based on sonification, that is, on the translation of movement parameters into the sound quality of a musical track. This translation has an appeal to the human reward system such that music-based reinforcement learning becomes possible. The system was compared to standard verbal instructions by an instructor. Both feedback types were able to improve the movement parameters with respect to an initial condition and were perceived equivalently clear and pleasurable by participants. The current findings suggest that such systems are a valuable addition to current training methods and more motivational versions could be developed to be used also in the fields of rehabilitation and medical treatments.
